# Commercial Bee Pollen with Different Geographical Origins: A Comprehensive Approach

**DOI:** 10.3390/ijms130911173

**Published:** 2012-09-07

**Authors:** Carla Nogueira, Antonio Iglesias, Xesus Feás, Leticia M. Estevinho

**Affiliations:** 1 CIMO-Mountain Research Center, Agricultural College of Bragança, Polytechnic Institute of Bragança, Campus Santa Apolónia, Bragança 5301-855, Portugal; E-Mail: carla.m.p.nogueira@hotmail.com; 2 Department of Anatomy and Animal Production, Faculty of Veterinary, University of Santiago de Compostela, Lugo 27002, Galicia, Spain; E-Mail: antonio.iglesias@usc.es; 3 Department of Organic Chemistry, Faculty of Sciences, University of Santiago de Compostela, Lugo 27002, Galicia, Spain; E-Mail: xesus.feas@usc.es

**Keywords:** bee pollen, microbiological characterization, physicochemical characterization, pollinic analysis, labeling rules

## Abstract

Since the primordial of humanity, pollen has been considered a good source of nutrients and energy. Its promising healing properties have also been referred to. The present study aimed to characterize, for the first time, eight commercial pollens from Portugal and Spain available on the market studying the legislation on labeling, pollinic origin, physicochemical and microbiological analyses and identification of yeasts. Eleven botanical families were found amongst the samples. The most abundant family and the most dominant pollen was *Cistaceae*. The moisture content, ash, *a_w_*, pH, reducing sugars, carbohydrates, proteins, lipids and energy were analyzed and the specific parameters were within the specifications required by some countries with legislation regarding these parameters. Microbiologically commercial pollen showed acceptable safety for the commercial quality and hygiene. All samples showed negative results for toxigenic species. The microorganisms studied were aerobic mesophiles, yeasts and moulds, coliforms, *Escherichia coli*, *Staphylococcus aureus*, *Salmonella* and sulfite-reducing *Clostridium*. During the work, six yeasts species were isolated from pollen, with *Rhodotorula mucilaginosa* being the most abundant, as it was present in four samples.

## 1. Introduction

Bee pollen, commonly designated as the “the life-giving dust”, results from the agglutination of flower pollen, nectar or honey and bees’ salivary substances. Indeed, this natural product is recognized to be a valuable apitherapeutic product with potential for medical and nutritional applications [1]. Bee pollen is promoted as a health food with a wide range of nutritional and therapeutic properties [2], triggering beneficial effects to human health and the prevention of prostate problems [3], allergy desensitization [4], arteriosclerosis [5] and tumors [6]. Its important physiological functions have already been widely praised. It has been reported that bee pollen accelerates mitotic rate, promotes tissue repair, enhances greater toxic elimination and reduces excessive cholesterol levels [7]. Its radical scavenging activity has already been reported [8].

This product is also used as a food supplement since it contains the richest known source of carbohydrates, proteins, amino acids, lipids, vitamins, minerals and trace elements [9], but also significant amounts of polyphenol substances, mainly flavonoids [10]. In fact, some nutritionists state that human beings could live adequately just by eating bee pollen [7].

However, it is important to note that from the anther dehiscence to comb cells, pollen is exposed to microbial contamination. This contamination can be attributed to various factors and sources, honeybees, weather, plant materials, insects and animals, humans and their agricultural devices [11]. In addition, when no prompt and adequate drying is performed by the beekeeper after collection by bees, bee pollen is a major substrate for mycotoxin growth and occurrence of undesirable fermentations [12]. Also, due to its high amount of proteins, if not stored correctly, it can lose the nutritional value quickly, undergoing Maillard reactions [1]. In this context, it is necessary to control and supervise the processes of preparation and storage, in order to ensure that the product supplies consumers with all the available nutrients, maintains its good organoleptic proprieties and has good microbiological quality. The last is of utmost importance, mainly when the bee pollen is intended for human consumption.

Despite the studies that have been conducted on the nutritional characterization and biological properties of bee pollen, the microbiological quality and safety has never been subjected to a comprehensive study. Also, the labels of the bee pollen flasks have never been analyzed and verified according to the specific legislation.

In this context, the present study aims to assess the chemical composition, pollinic profile, microbiological quality, safety and the labeling of eight commercial bee pollens with various botanical and geographical origins. As far as we know, this is the first study about commercial pollen.

## 2. Results and Discussion

### 2.1. Labeling and Information to Consumers

Consumers should be able to be confident with their choice of foods and be able to buy according to their particular requirements, be it for diet and health, personal taste and preferences, or cost. All the essential information about the product must be visible in the labeling. With the aim of guaranteeing food authenticity and avoid misdescription, the labeling of the purchased samples was verified according to the specific legislation. In Table 1 the results of the labeling analysis are presented.

The mandatory items [13], which should appear on the product label, are the list of ingredients, the sale name, the net quantity in kilograms or grams and the date of minimum durability. Most still present the company name, address of the manufacturer, packer or seller, established within the EU, special storage conditions, the place of origin or provenance and the batch preceded by the letter “L”. All the products (100%) had the sales name, date of minimum durability, lot and net quantity. Regarding the country of origin of the pollen and indication of trade name or company name of producer/packer/seller established within the European Union, only one of the packages did not present this information, and the remaining 87.5% had these indications.

The pollen samples B, C, D and G are from Portugal, the samples E, F and H are from Spain and sample A had no indication of country of origin. The address of the manufacturer, packer or seller established within the European Union is not indicated in 12.5% of the packaging. Seventy-five percent 75% of packaging presented other consumer information that is not mandatory. Not obligatory information includes the storage conditions. Information about the effect of carbohydrates, amino acids, enzymes, vitamins and minerals in the organism was found in 37.5% of the packaging. The nutritional information was present in 62.5% and consumer information line was indicated in 37.5% of the packaging. The method of use was designated in 62.5% of the analyzed containers.

### 2.2. Pollinic Identification

The results of the pollen analysis are presented in Table 2. The bee pollens’ pollinic profile allows scientists to infer the vegetation present in the area and to date and ascertain any biodiversity changes, as for example, the presence and distribution of invasive or exotic plants. From the pollen analysis it can be seen that all samples were heterofloral. In addition, due to the different colors of the pollen grains it can be concluded that the samples included different botanical species.

Eleven families were found in the samples, with *Cistaceae* being the dominant in the three samples A (68.2%), C (83.6%) and E (90.6%) and type *Fabaceae* in two of the samples B (56%) and F (69.8%). The types *Ericaceae*, *Fagaceae*, *Boraginaceae* were dominant in D (58%), G (45.4%) and H (49.6%), respectively. The type *Boraginaceae* (*Echium*) was present in all the analyzed samples, apart from Pollen D. Indeed, this botanical family is present in several regions of Portugal and Spain and has several utilizations in cosmetics and as a functional food [14]. These applications are related with its high levels of alpha linolenic acid (ALA), gamma linolenic acid (GLA) and stearidonic acid (SDA). In this study four types of pollen belonging to different families were also found that were only present in small amounts (accessory pollen and isolated pollen). This is the case for *Pinus* spp., found in Sample B (12.4%), *Eucalyptus* spp., found in Sample G (9%), *Mimosa* spp., found in Sample D (10%) and *Quercus* spp., found in Pollen G (16%).

None of the botanical families is represented in all the samples studied, since bee pollen can vary according to the region, a factor which depends on the available surrounding bee pasture in the apiary vegetation, as well as on the climate conditions for flowering [15].

### 2.3. Chemical Composition

The chemical composition of the samples is shown in Table 3. The composition of pollen showed variations between samples, which may be due to the different botanical composition, geographical origin of the product, influence of age and environmental conditions [16].

The water content (expresses the total content: the free and bound water) and the water activity (measures the amount of free water) play an important role in the organoleptic characteristics and “shelf lifetime” of bee pollen [17]. When its values are too high, it can potentially promote microbial contamination, mainly by moulds and yeasts [18]. The moisture content varied between 8.40 ± 0.80% (G) and 6.02 ± 0.18% (C, D). The pollen’s humidity was above the upper limit (4%) set by the Brazilian legislation for commercial pollen [19], but all values were within the limits allowed in Argentina and Switzerland (8% in both) [20], except for pollen G (8.40 ± 0.82). However, the last was within the established limits of Bulgaria (10%) for commercial bee pollen. Pollen with less than 3% of moisture is undesirable since it can result in discoloration and development of chemical reactions (for example, the Maillard reaction and lipid oxidation), resulting in undesirable odors and a rancid product [21]. The water activity was higher in sample A (0.43 ± 0.025) and lower in sample D (0.26 ± 0.01) and there were no statistically significant differences (*p* < 0.001). These results are similar to those reported by [22] (*a_w_* ranged from 0.32 to 0.55), who studied bee pollen provided by beekeepers. All samples showed low values of *a_w_*, which is characteristic of dehydrated foods, allowing storage stability. Indeed, since pollen is hygroscopic, high values of *a_w_* promote microbiological contamination, more precisely by yeasts and fungi, which produce mycotoxins and ochratoxins, creating a risk to the consumer [23].

The pH and *a_w_* have great importance during the storage of bee pollen, as they influence its texture, stability and shelf life [24]. The pH of the commercial pollens ranged from 5.17 ± 0.09 (D) to 4.23 ± 0.14 (G), the samples B, C and E did not differ significantly from each other (*p* < 0.001). The values are in accordance with the Brazilian law (4.0 to 6.0) and those published by [16,17,22,25,26].

The ash content is an account of the inorganic matter present in bee pollen [15]. For the ash content the values ranged from 0.5 ± 0.01 (H) to 3.16 ± 0.03 (A). The pollen H was statistically significantly different from the others (*p* < 0.001). Of the eight samples, seven are within the range reported by [26], who analyzed bee pollen from Brazil, and [17], who studied pollen from Argentina. Some values were lower than those reported by [10], who found values between 1.90 and 3.91% for Spanish bee pollen. The ash content is influenced by soil type, geographical origin, flora species and capacity of the plant to accumulate minerals [21]. The presence of mineral impurities is due to inefficient cleaning procedures and may increase the ash content, which makes this analysis an important quality index for pollen [10].

Some authors emphasize the value of pollen as an important source of proteins [10]. The protein content showed statistically significant differences (*p* < 0.001), for pollen H, with values ranging from 25.15 ± 1.64 (C) to 12.50 ± 0.58 (H). The great variability of protein content found in bee pollen can be partly explained by the natural variation of the composition that is a consequence of the distinct source plants. In addition, differences within the same plant species growing under different environmental conditions have also been reported [1]. Our results were similar to those obtained by [1,26,27] in pollen samples from Brazil. Lower values for pollen from Spain were found by [10]. However, higher values have already been obtained. The results of [28,29] who analyzed samples from Brazil and Argentina, ranged from 23.59% to 27.70% and 24.17% to 37.32%, respectively. The nitrogen content of Portuguese organic pollen was between 34.00% and 24.23% [22], suggesting that this product is of better quality.

Lipids, mainly the fatty acids esters, are partly responsible for the physicochemical properties [30]. The fat content varied from 3.06 ± 0.08 (G) to 2.35 ± 0.11 (B) with no statistically significant differences (*p* < 0.001) between all pollens apart from B (*p* < 0.001). These values are similar to those reported by [17,22] and slightly inferior to those obtained by [1,19,26].

The higher carbohydrate content, 84.25 ± 0.58, was observed in pollen H, while pollen Sample C (69.68 ± 2.08) showed the lowest level, the latter showing statistically significant differences (*p* < 0.001). The values are within the limits described by [10], but the values obtained by [27] are superior to ours. The values obtained for energy were between 400.70 ± 0.36 and 411.75 ± 0.63 (kcal/100 g) with no statistically significant differences (*p* < 0.001) among themselves for Pollen B, C, D, E and F.

The reducing sugar content was highest in Pollen E (41.79 ± 3.21) and smallest in Sample A (26.09 ± 3.60). The high reducing sugar content of bee pollen has been associated with the presence of nectar/honey, commonly used by bees as glue for the pollen of plants [27].

### 2.4. Microbial Contamination

The pollen contamination by microorganisms is dependent on the nutritional composition as well as the harvesting practices, cleaning, drying and storage of the product. The results obtained for the levels of microbial contamination are shown in Table 4.

The results obtained for the parameters that indicate commercial quality are in agreement with the Argentinian Food Code, which establishes the maximum of 1.5 × l0^3^ ufc/g for moulds and yeasts. In this study, moulds and yeasts were detected in 50% of the samples, while [17,31] found it in all the samples. [22] detected these microorganisms in 60% of the samples, however, the number per sample was lower. Aerobic mesophiles were found only in 12.5% of the samples, and the number of colony forming units oscillated between <10 and 8.7 × 10^3^ ± 7.8 × 10. Sample F was significantly different from the others (*p* = 0.032). The number of colony forming units of moulds, yeasts and aerobic mesophiles found by [22] in non-commercial Portuguese bee pollen was inferior. The higher number of microorganisms found in the present study must be related to the higher content of water of the commercial pollen.

The indicators of safety (*Salmonella* sp., sulfite-reducing *clostridia*) and the indicators of sanitary quality (fecal coliforms, *Escherichia coli* and *Staphylococcus aureus*) were absent. These results corroborate the observations of [22]. On the other hand, [17] fecal coliforms were found in some samples.

### 2.5. Isolation and Identification of Yeasts

**Figure 1:**
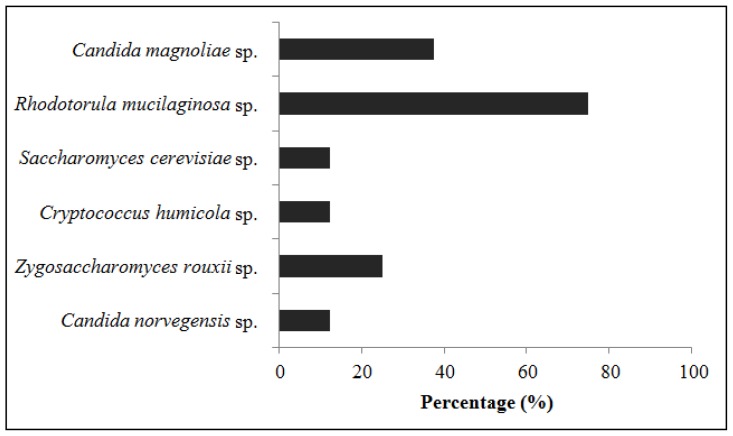
Percentage of the yeasts isolated from the samples.

This study is the first to identify the yeasts present in bee pollen. Six yeast species were identified in the samples in which yeasts were initially found (Figure 1).

Two of the six species found belong to the genus *Candida* (*norvegensis* and *magnoliae*). The yeast *Rhodotorula mucilaginosa* sp. was the most frequent. It was present in 75% of the samples with yeasts. *Candida magnoliae* sp. and *Zygosaccharomyces rouxii* sp. were identified in 37.5% and 25% of the samples, respectively. *Candida norvegensis* sp., *Cryptococcus humicola* sp. and *Saccharomyces cerevisiae* sp. were found in 12.5% of the samples. Studies on the yeasts isolated from honey have revealed that the most common is *Candida magnoliae* sp., followed by *Rhodotorula mucilaginosa* sp. [32]. These results corroborate the claim that the microorganisms present in honey are, essentially, from pollen [33]. *Rhodotorula mucilaginosa* is a common environmental inhabitant. This microorganism may act as an indicator of a lack of hygiene standards [34]. To the best of our knowledge, *Candida magnoliae*, that is commonly isolated from the honeycomb, is inoffensive. It has been implicated in just one case of human disease (tenosynovitis in an immunocompetent child) [35]. *Saccharomyces cerevisiae* and *Zygosaccharomyces rouxii* are responsible for the deterioration of foods that were processed and packaged in accordance with normal standards of good manufacturing practice.

*Candida norvegensis* sp. and *Rhodotorula mucilaginosa* sp. can cause health risks, colonize the skin, fingernails, respiratory tract, genital-urinary and cause diarrhea.

These results indicate that the optimization of the hygienic procedures during all the production chain is necessary to improve the quality and safety of the product. In order to reduce the risk of food-borne illness and spoilage, good practices in agriculture (GAP), hygiene (GHP) and manufacturing (GMP) must be implemented.

## 3. Experimental Section

### 3.1. Samples

**Figure 2:**
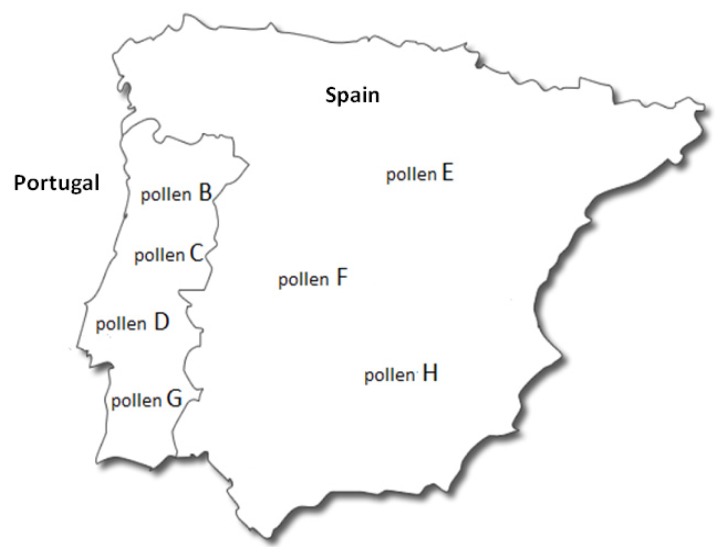
Place of origin of the commercial bee pollen. The origin of Sample A was not specified in the labeling.

Eight commercial pollens (A-H) of different floral sources and geographical origins (Figure 2) were purchased from the market and left in the dark at room temperature (± 20 °C) until further analysis.

### 3.2. Chemicals and Materials

The sodium sulfate (Na_2_SO_4_), anhydrous, powder, extra pure, sulfuric acid (H_2_SO_4_) and the sodium hydroxide (NaOH) were purchased from Acros Organic (Geel, Belgium). The petroleum ether and ethanol were obtained from Sigma Chemical Co. (St. Louis, MO, USA). The culture mediums were purchased from Himedia (India). The water was purified using a Milli-Q purification system (Millipore, Bedford, MA, USA).

### 3.3. Labeling

The labeling of the pollen bought at the supermarket was analyzed in accordance with the requirements of current Portuguese law [13].

### 3.4. Pollinic Identification

The pollinic identification was performed by the method described by [1]. Briefly, a sample of 2 g, corresponding to more or less 300 pollen pellets, was considered to be representative for botanical origin. The pellets were then divided into sub-samples according to color (yellow-black, dark brown, green, red, yellow and orange). Each subsample was then weighed to calculate its percentage of the whole sample. The sample was placed between blade and slide using glycerin gel as mounting medium. Identification of pollen grains was performed by optical microscope with total magnification (400× and 1000×). A reference collection of CIMO-Mountain Research Centre (Agricultural College of Braganza) and guides of pollen morphology were used for the recognition of the pollen types. The following terms were used for pollen frequency classes: dominant pollen (DP, more than 45% of the pollen grains counted), accessory pollen (AP, 15%–45%) and isolated pollen (IP, less than 15% of the pollen grains counted) [36].

### 3.5. Physicochemical Analyses

All physicochemical tests were performed in triplicate.

#### 3.5.1. Moisture

Approximately 3 g of each sample of the study pollen was dried at a temperature of 100 °C–105 °C to constant weight [37].

#### 3.5.2. Ash

The ash content was determined after ignition at 600 ± 15 °C by gravimetry, as reported by [19].

#### 3.5.3. pH

The pH was measured in the aqueous phase obtained after mixing 10 g of pollen in 75 mL of distilled water [37].

#### 3.5.4. Reducing Sugars

With the aim of performing the acid hydrolysis of the reducing sugars, 60 mg of each pollen was dissolved in a H_2_SO_4_ solution (10 mL, 1.5 M). The solutions were heated in a water bath (100 °C) for 20 min. Afterwards, the solution was cooled, neutralized with 12 mL NaOH (10%, *w*/*v*), filtered and the volume of the flask (60 mL) was completed with distilled water. The quantification of the reducing carbohydrates was performed spectrophotometrically at 540 nm using a spectrophotometer (UV-VIS spectrometry Unicam Hekios, UK). Glucose was used as standard. For this, 2 mL of distilled water, 1 mL of each hydrolyzate sample and 1 mL of DNS were placed in a test tube. The mixture was heated for 5 min in a water bath (100 °C) and cooled rapidly on ice to room temperature. Next, 5 mL of distilled water was added and the absorbance was read [38].

#### 3.5.5. Water Activity

This parameter was determined by placing each pollen sample directly into a water activity meter (Rotronic HygroPalm. Bassersdorf CH-8303).

#### 3.5.6. Lipids

Two grams of pollen were macerated in a mortar with anhydrous Na_2_SO_4_ by detachment of the mixture. Then, it was extracted with *n*-hexane for about 4 h in the Soxhlet apparatus [19].

#### 3.5.7. Proteins

Nitrogen content was determined using the Kjeldahl method (230-Hjeltec Analyzer, Foss Tecator, Höganäs, Sweden). The crude protein (CP) content was calculated using the conversion factor of 6.25 (*N* × 6.25). The crude fat (CF) was determined by gravimetry after extraction with petroleum ether using an automatic Soxtec device (FOSS, Soxtec™ 2050, Höganäs, Sweden).

#### 3.5.8. Carbohydrates

The total carbohydrate contents was obtained by difference [100 − (ashes + proteins + lipids)] (%) [36].

#### 3.5.9. Energy

The total energy (in kcal) was estimated according to the following equation: energy (kcal) = 4 × (g protein+ g carbohydrate) + 9 × (g lipid) [39].

### 3.6. Microbiological Determinations

To assess the microbiological quality of the samples, the commercial quality parameters (mesophilic microorganisms, yeasts and moulds), the indicators of sanitary quality (fecal coliforms and *Escherichia coli*) and the indicators of safety (sulphite reducing clostridium spores and *Salmonella*) were analyzed. All microbial tests were performed in triplicate.

#### 3.6.1. Sample Preparation

Sample preparation was carried out according to [40]. Ten grams of the sample of pollen were weighed on a scale (Mettler PC Model 2000), and homogenized with 90 mL of Ringer’s solution (dilution 10^−1^). For each sample, decimal dilutions were prepared using as diluent successive Ringer’s solution.

#### 3.6.2. Enumeration of the Total Mesophilic Microorganisms

Enumeration was made on Plate Count Agar (Himedia), incubated at 37 °C for 48 h [41]. Microbial counts were expressed as colony-forming units per gram of bee pollen (cfu/g).

#### 3.6.3. Enumeration of Yeast and Moulds

Mould and yeast enumeration was made on DG18 (Himedia) and incubated at 25 °C for 5 days [42]. Microbial counts were expressed as colony-forming units per gram of bee pollen (cfu/g).

#### 3.6.4. Sulfite Reducing Clostridium Spores

This analysis includes three steps, according to the protocol of [43]: preparation, inoculation and enumeration. For sulfite-reducing clostridia counting, aliquots of 10, 5, 1 and 0.1 mL of the initial suspension were added to an empty tube, thermally treated at 80 °C for 15 min and covered with Differential Reinforced Clostridial Broth (DRCM) (Himedia), and incubated at 37 °C for 5 days. At the end, the black colonies were counted. The results are expressed as presence of sulfite-reducing Clostridia in 0.01 g. Results were expressed as most probable numbers of sulfite-reducing Clostridium spores per gram of bee pollen (MPN/g).

#### 3.6.5. Fecal coliforms and *Escherichia coli*

The presence of coliforms, fecal coliforms and *E. coli* in bee pollen may be determined by means of the Most Probable Number (MPN) procedure, as described in [44]. Briefly, this method involves serially diluting out the target organisms in the sample, in five replicate aliquots, to extinction. The probable level of the target organisms is then statistically estimated from a Hoskins table. Results were expressed as the most probable numbers of coliforms per gram of bee pollen (MPN/g) and the most probable numbers of *E. coli* per gram of bee pollen (MPN/g).

#### 3.6.6. Salmonella

The detection was performed following the protocol of [45]. According to this protocol, the search of *Salmonella* has four steps that include pre-enrichment (Bulfered Peptone Water), selective enrichment (Rappaport Vassiliadi and Muller-Kauffmann Tetrathionate-Novobiocin Broth-MKTTn), planting out (Xylose lysine Deoxycholate Agar-XLD agar) and confirmation Nutrient Agar (NA). Afterwards, biochemical confirmations are performed. Results were expressed as absence or presence of *Salmonella* in 25 g of bee pollen.

### 3.7. Isolation and Identification of Yeasts

The API 20C AUX system, a commercial kit for the evaluation of the assimilation of 19 carbon sources, was used according to the instructions in the identification of the isolated yeasts. Pure cultures of each test organism were grown on one plate each of Sabouraud agar for 48 h. Each strain was inoculated into 2 mL of a NaCl solution (0.85%, *w*/*v*) to achieve a turbidity visually equivalent to a 2 McFarland turbidity standard. One 100 μL aliquot of this preparation was inoculated into the corresponding C medium tube and each tube of inoculated medium was transfer-pipetted into the corresponding API 20C AUX yeast panel. Each panel was incubated at 30 ± 2 °C for 48 h. All panels were read for growth according to the API 20C AUX-analytical profile index.

### 3.8. Statistical Analysis

Results are shown as mean values and standard deviation. Prior to the statistical analysis, normality tests (Shapiro-Wilk, Anderson-Darling, Liliefors and Jarque-Bera) were carried out. In each parameter, the differences between pollens were analyzed using one-way analysis of variance (ANOVA) followed by Tukey’s HSD Test with *α* = 0.05. This treatment was carried out using SAS v. 9.1.3 program.

## 4. Conclusions

This work is a comprehensive survey about the physicochemical composition, pollinic spectrum and microbiological safety of commercial bee pollen from different origins. In parallel the specific legislation of the bee pollens concerning the labeling rules was also verified. The physicochemical parameters (water content, *a_w_*, pH, ash, proteins, lipids, carbohydrates, reducing sugars and energy) of the samples were within the specifications. The indicators studied suggest that the commercial pollen is safe from a microbiological standpoint. Concerning the labeling, all the products presented the sales name, date of minimum durability, lot and net quantity.

Bee pollen has promising therapeutic and nutritional applications and, as such, the investigation of its chemical, nutritional and microbiological composition is a sound research priority.

## Figures and Tables

**Table 1:** Results of the commercial bee pollen labeling analysis.

	In the packaging (%)
Trade name	100.0
Net quantity (g or kg)	100.0
Date of minimum durability	100.0
Name, company or business name	87.5
Social grower/packer/seller	87.5
established in the EU	87.5
Lot	100.0
Address	87.5
Place of origin	87.5
* Storage conditions	75.0
* Information to consumers	75.0

* Items not required on labels.

**Table 2:** Palynological spectrum of the eight commercial bee pollen (mean ± standard deviation; *n* = 3).

Family	Samples								

		A	B	C	D	E	F	G	H
Asteraceae	Leontodon spp. (%)	-	5.3 ± 1.9 3	-	-	1.2 ± 0.1 3	-	18.2 ± 3.4 2	-
Boraginaceae	Echium spp. (%)	2.0 ± 0.4 3	4.6 ± 1.4 3	7.5 ± 1.6 3	-	4.2 ± 0.8 3	1.2 ± 0.2 3	6.2 ± 2.3 3	49.6 ± 5.2 1
Cistaceae	Cistus spp. (%)	68.2 ± 8.9 1	-	83.6 ± 9.2 1	2.4 ± 0.5 3	90.6 ± 9.3 1	-	5.2 ± 1.5 3	-
Ericaceae	Erica spp. (%)	-	-	-	58.0 ± 7.7 1	1.7 ± 0.2 3	-	-	-
Fabaceae	Cytisus spp. (%)	-	57.0 ± 9.2 1	2.9 ± 0.3 3	3 ± 0.8 3	-	69.8 ± 6.7 1	-	8 ± 1.0 3
	Acacia spp. (%)	-	2.5 ± 0.6 3	-	-	1.5 ± 0.3 3	-	-	-
	Trifolium spp. (%)	4.4 ± 1.2 3	-	4.8 ± 1.0 3	16.3 ± 2.4 2	-	-	45.4 ± 7.1 1	2.0 ± 1.0 3
Fagaceae	Quercus spp. (%)	-	-	-	-	-	-	16.0 ± 0.8 2	-
	Castanea spp. (%)	15.8 ± 3.2 2	13.8 ± 2.3 3	1.2 ± 0.3 3	-	-	24.8 ± 5.1 2	-	5.2 ± 1.3 3
Lamiaceae	Thymus spp. (%)	-	-	-	10.3 ± 3.9 3	-	1.4 ± 0.4 3	-	9.0 ± 2.1 3
Mimosaceae	Mimosa spp. (%)	-	-	-	10.0 ± 3.6 3	-	-	-	-
Myrtaceae	Eucalyptus spp. (%)	-	-	-	-	-	-	9.0 ± 1.9 3	-
Pinaceae	Pinus spp. (%)	-	12.4 ± 2.7 3	-	-	-	-	-	-
Rosaceae	Prunus spp. (%)	5.0 ± 1.6 3	4.4 ± 0.9 3	-	-	0.8 ± 0.2 3	2.8 ± 0.4 3	-	-
-	Rubus spp. (%)	4.6 ± 1.3 3		-				26.2 ± 4.3 2	

1 DP—Dominant Pollen (>45%);

2 AP—Accessory Pollen (15%–45%);

3 I—Isolated Pollen (<15%).

**Table 3:** Proximate chemical composition (g/100 g of fresh weight) and energetic value (kcal) of the eight bee pollen samples (mean ± standard deviation; *n* = 3).

	A	B	C	D	E	F	G	H
Water content	7.63 ± 0.27 ab	7.91 ± 0.04 ab	6.02 ± 0.07 c	6.02 ± 0.18 c	7.97 ± 0.07 a	6.95 ± 0.39 bc	8.40 ± 0.82 a	7.82 ± 0.25 ab
Water activity	0.43 ± 0.03 a	0.35 ± 0.02 cd	0.33 ± 0.01 d	0.26 ± 0.01 e	0.41 ± 0.004 ab	0.33 ± 0.02 d	0.40 ± 0.03 ab	0.38 ± 0.017 bc
pH	4.92 ± 0.02 ab	4.62 ± 0.04 c	4.71 ± 0.03 bc	5.17 ± 0.09 a	4.49 ± 0.14 cd	4.99 ± 0.17 ab	4.23 ± 0.14 d	5.03 ± 0.10 a
Ash	3.16 ± 0.03 a	1.85 ± 0.05 cd	2.38 ± 0.39 bc	2.23 ± 0.03 bcd	2.16 ± 0.29 cd	2.83 ± 0.29 ab	1.67 ± 0.29 d	0.5 ± 0.01 e
Proteins	20.84 ± 1.16 ab	20.03 ± 1.49 b	25.15 ± 1.64 a	22.01 ± 1.43 ab	22.45 ± 0.89 ab	20.69 ± 2.64 b	18.79 ± 1.62 b	12.50 ± 0.5 c
Lipids	2.66 ± 0.06 c	2.35 ± 0.11 d	2.79 ± 0.10 bc	2.71 ± 0.03 c	2.86 ± 0.09 bc	3.33 ± 0.16 a	3.06 ± 0.08 ab	2.75 ± 0.13 c
Carbohydrates	73.35 ± 1.21 bc	75.76 ± 1.48 b	69.68 ± 2.08 c	73.06 ± 1.39 bc	72.53 ± 1.04 bc	73.15 ± 2.88 bc	76.49 ± 1.95 b	84.25 ± 0.58 a
Reducing sugars	26.09 ± 3.60 c	33.19 ± 2.36 abc	35.68 ± 5.07 abc	38.62 ± 2.19 ab	41.79 ± 3.21 a	34.52 ± 1.05 abc	26.90 ± 3.30 c	29.39 ± 4.71 bc
Energy kcal	400.70 ± 0.36 d	404.37 ± 0.59 c	404.43 ± 1.10 c	404.66 ± 0.03 c	405.66 ± 1.51 c	405.30 ± 1.73 c	408.62 ± 1.06 b	411.75 ± 0.63 a

The letters (a, b, c) represent which bee pollens are different by Tukey test with a significance of *p* = 0.05.

**Table 4:** Microbial analyses of eight commercial bee pollen samples (mean ± standard deviation; *n* = 3).

	A	B	C	D	E	F	G	H	*p*-Value
Aerobic mesophiles (cfu/g)	<10 a	<10 a	<10 a	<10 a	<10 a	8.7 × 10^3^ ± 7.8 × 10 b	<10 a	<10 a	0.032
Moulds and yeasts (cfu/g)	<10 a	<10 a	<10 a	8.8 × 10^2^ ± 6.9 × 10 b	< 10 a	9.4 × 10^2^ ± 7.1 × 10 b	2.6 × 10^2^ ± 5.2 × 10 ab	6.9 × 10^2^ ± 4.4 × 10^1^ b	0.001
Fecal coliforms (MPN/g)	<1	<1	<1	<1	<1	<1	<1	<1	n.a.
*Escherichia coli* (MPN/g)	Absent	Absent	Absent	Absent	Absent	Absent	Absent	Absent	n.a.
Sulphite reducing clostridium (MPN/g)	Absent	Absent	Absent	Absent	Absent	Absent	Absent	Absent	n.a.
*Salmonella* sp. (in 10 g)	Absent	Absent	Absent	Absent	Absent	Absent	Absent	Absent	n.a.

The letters (a, b) represent which honeys were different by Tukey test with a significance of *p* = 0.05.
